# COVID-19: Presumed Infection Routes and Psychological Impact on Staff in Administrative and Logistics Departments in a Designated Hospital in Wuhan, China

**DOI:** 10.3389/fpsyg.2020.01501

**Published:** 2020-06-12

**Authors:** Li-Sha Luo, Ying-Hui Jin, Lin Cai, Zhen-Yu Pan, Xian-Tao Zeng, Xing-Huan Wang

**Affiliations:** ^1^Center for Evidence-Based and Translational Medicine, Zhongnan Hospital of Wuhan University, Wuhan, China; ^2^Center for Evidence-Based and Translational Medicine, Wuhan University, Wuhan, China; ^3^Institute of Hospital Management, Wuhan University, Wuhan, China; ^4^Division of Medical Affairs, Zhongnan Hospital of Wuhan University, Wuhan, China; ^5^Division of Personnel Services, Zhongnan Hospital of Wuhan University, Wuhan, China

**Keywords:** COVID-19, SARS-CoV-2, nosocomial infection, staff, administrative and logistics departments, psychological intervention

## Abstract

**Objective:** Our aim was to explore the presumed infection routes and psychological impact of COVID-19 on staff in administrative and logistics departments (ALDs).

**Methods:** We gathered data from all 18 staff members with COVID-19 in ALDs in Zhongnan Hospital of Wuhan University, China. The baseline, job before diagnosis, presumed infection environment, use of protective equipment, and psychological status before and after diagnosis were collected and analyzed. A total of 18 uninfected staff members working alongside them in the same environment and 18 random matched infected doctors and nurses formed two control groups; the psychological impact of these three groups was then compared.

**Results:** Of the 18 members of staff, 88.89% were infected due to the working environment (hospital), and nine had face-to-face conversations with doctors and nurses in their daily work. Many staff members did not take any protective measures in their routine work. Before they were diagnosed, 12 staff members were aware of the seriousness of the epidemic, and most of the staff maintained a neutral attitude to the COVID-19 outbreak. A total of 77.78% of the staff experienced psychological stress or emotional changes after diagnosis, which were mainly caused by family health and disease related issues. Most of them managed their emotions by self-control and video calls with their families. There was no significant difference in psychological impact among the three groups, but uninfected staff members were fully aware of the seriousness of the epidemic.

**Conclusions:** Effective protective measures should be taken for staff members in ALDs. Psychological interventions are very important to help infected staff members in ALDs cope with psychological distress.

## Introduction

The novel coronavirus disease (COVID-19), caused by severe acute respiratory syndrome coronavirus 2 (SARS-CoV-2), has been spreading rapidly worldwide, creating a tremendous public health burden (Li et al., 2020). As of February 11, 2020, there were a total of 1,716 infected healthcare staff members (63% in Wuhan) (Wu and McGoogan, 2020). Staff in administrative and logistics departments (ALDs) are also front-line workers, alongside doctors and nurses, who provide strong support for the orderly conduct of medical work. Compared with doctors and nurses, these staff members receive less attention from society. Through their work in hospitals, this group is likely to be directly or indirectly exposed to the SARS-CoV-2 with a high risk of infection. Additionally, they suffered high psychological pressures from an increased workload, fears of possible infection of their families and colleagues, and a lack of knowledge about protection from infectious diseases (Lai et al., 2020).

Public health emergencies can easily cause anxiety and panic among healthcare workers, and previous studies have shown that the severe acute respiratory syndrome (SARS) outbreak has had adverse psychological effects on healthcare workers (Bai et al., 2004). The incidence of stress disorder among doctors and nurses has reached 27.39% during the COVID-19 epidemic (Huang et al., 2020a). Unlike doctors and nurses, staff in ALDs lack knowledge of protection, diagnosis, and treatment; the psychological effects of the epidemic may thus be more serious, especially for those contracting the disease. Therefore, identifying their presumed routes of infection and psychological changes is also crucial to the success of fighting COVID-19. Several studies about psychological effects on healthcare workers during the COVID-19 outbreak have been published, but none of them have focused on infected ALD personnel (Jin et al., 2020a; Kisely et al., 2020). The current study thus aimed to explore the potential infection routes and psychological changes among hospital staff in ALDs and to provide scientific suggestions on preventing adverse effects among this population's during large-scale infectious diseases outbreaks.

## Methods

### Study Design

This retrospective study was conducted in the Zhongnan Hospital of Wuhan University, one of the key hospitals at the epicenter of COVID-19 outbreak. The participants were confirmed as COVID-19 based on the diagnostic criteria of the National Health Committee of the People's Republic of China (Jin et al., 2020b; Ma et al., 2020). This study is part of a larger cross-sectional study and was reviewed and approved by the Committee for Ethical Affairs of Zhongnan Hospital (Approval number: 2020036). The study period was from February 15 to 29, 2020; and data about doctors and nurses have been published elsewhere (Jin et al., 2020a).

### Measuring Instruments and Data Collection

Data were collected using a validated electronic questionnaire, including informed consent, which was jointly developed by experts from multidisciplinary fields, such as epidemiology, evidence-based medicine, and front-line clinicians during the COVID-19 epidemic. The readability and content validity of the questionnaire were tested by experts from several fields from different medical institutions, and the test-retest reliability was 0.82 (Wang et al., 2020a,b). The questionnaire items included basic information, exposure history, protective measures, clinical symptoms, treatment measures, and psychological changes. In terms of psychological items, we collected staffs' awareness and feelings about the epidemic before diagnosis, as well as their psychological changes and coping mechanisms after diagnosis, to get a preliminary understanding of the impact of the epidemic on ALD staffs. All 18 infected staff members in the ALDs of this hospital were contacted through the Division of Medical Affairs. To ensure the accuracy of results, we confirmed the exposure status through phone calls to all participates and their department directors.

Additionally, we compared the psychological impact between infected staff in ALDs with two control groups: one was 18 infected doctors and nurses, randomly selected from the 103 infected staff members (Jin et al., 2020a), and the other group was uninfected ALDs staff members, nominated by their infected colleagues who worked in the same environment. They were also investigated used the validated electronic questionnaire (Wang et al., 2020b).

### Statistical Analysis

Categorical variables were described as counts and percentages; Wilcoxon signed rank sum tests and Fisher exact tests were conducted to compare the psychological impact between staff in ALDs and the two control groups. The data analysis was performed by the SAS software, version 9.4 TS1M6 (SAS Institute Inc., Cary, NC) and visualized by Microsoft PowerPoint 2016, where *P* < 0.05 was considered statistically significant.

## Results

### Basic Information

All 18 staff members with COVID-19 in ALDs were included in this study (Table 1), and all have now recovered. Five were males and 13 were females, and their ages ranged from 28 to 59 years. A total of 88.89% thought they were infected by the working environment in hospitals, and one case did not know the source of infection. Nine staff members regularly had face-to-face conversations with doctors and nurses in the course of their work. More than half of staff thought that the way they got the infection was droplets and contact transmission.

**Table 1:** Characteristics of COVID-19 patients among staff members in administrative and logistics departments.

**Departments**	**Male/** **Female**	**Age** **(years)**	**Job before diagnosis**	**Infectious environment**	**Protective equipment**		**Condition**	**Clinical outcome**
					**Masks**	**Gloves**		
Security	2/1	43	Distributing medical materials	Hospital	✓✓✓✓	✓✓	2 Mild, 1 Moderate	Recovered
Finance	1/0	28	Dealing with staff' financial reimbursement	Hospital	✓✓✓	✓✓	Moderate	Recovered
CPC organization	0/1	37	Managing the work of cadres	Hospital	✓✓✓	✓✓✓	Moderate	Recovered
Logistics Support (Cleaning)	1/3	45	Environmental cleaning	3 in hospital, 1 unclear	✓✓✓	✓✓✓	1 Mild, 3 Moderate	Recovered
Logistics Support (Elevator operation)	0/1	51	Operating elevator	Hospital	×	×	Moderate	Recovered
Scientific Research	0/1	35	Managing laboratory	Other	✓	✓✓✓	Moderate	Recovered
Convalescent	1/0	59	Accompanying healthcare staff to patient's home	Hospital	×	×	Moderate	Recovered
Personnel Services	0/1	48	Receiving and handling staff' promotion materials	Hospital	✓✓✓	✓	Moderate	Recovered
Sterilized Supplying	0/2	52	Transporting patients, cleaning surgical instruments	Hospital	✓✓✓✓	✓✓✓✓	Moderate	Recovered
Medical Insurance	0/1	34	Submitting reports, handling medical insurance problems for patients	Hospital	✓✓✓	×	Moderate	Recovered
Nosocomial Infection	0/1	46	Routine works	Hospital	✓✓✓✓	✓✓✓	Moderate	Recovered
Operations Management	0/1	31	Calculating staff performance	Hospital	×	×	Moderate	Recovered

*✓✓✓✓, always wear protective equipment, ✓✓✓, often; ✓✓, sometimes; ✓, occasionally; × , never*.

### Presumed Infection Routes

Table 1 presents detailed information of these 18 staff; four who worked as hospital environmental cleaners and often wore masks and gloves during work, while another one, responsible for operating the elevator, never wore masks or gloves. Three staff members in the Security Department who distributed medical materials to each department sometimes wore gloves. Two staff working in the Sterilized Supply Center transporting patients and cleaning surgical instruments always wore masks and gloves. One person in Convalescent Department who was responsible for accompanying doctors to patients' homes never wore masks and gloves at work. One person working in the Division of Operation Management to calculate the hospital's performance never took any protective measures. One person in the Division of Medical Insurance never wore gloves when handling insurance problems for patients. One person in the Division of Personnel Services occasionally wore gloves when receiving documents from staff. One person working in the Scientific Research Center as laboratory manager occasionally wore masks.

### Psychological Status

**Figure 1:**
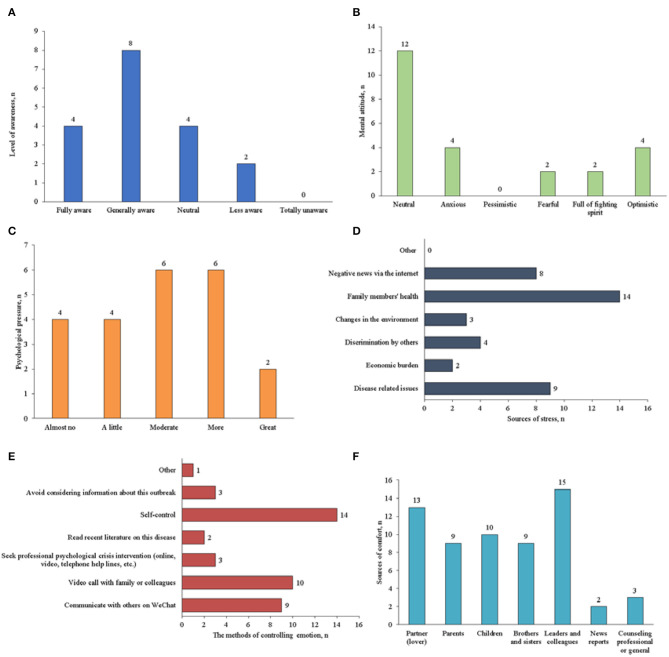
Psychological status before and after diagnosis of the COVID-19 staffs in administrative and logistics departments (**A**: awareness of the epidemic before diagnosis; **B**: mental attitude before diagnosis; **C**: psychological stress or emotional changes after diagnosis; **D**: the possible causes of emotional change after diagnosis; **E**: the methods used to control stress or mood changes after diagnosis; and **F**: the sources of comfort and care after diagnosis).

The psychological status before and after diagnosis of these infected staff were shown in Figure 1. Before they were diagnosed, 12 staff said they were aware of the seriousness of the epidemic. Most staff's attitude remained neutral to COVID-19 outbreak, and none of them were pessimistic. During the treatment, 77.78% of staff experienced psychological stress or emotional changes, which were mainly caused by family health, disease related issues and negative news via the internet. They managed their emotions and stress by self-control, video calls with family members or colleagues, and communicating with others on WeChat. Most staff received comfort and care from leaders and colleagues, partners, and children.

Additionally, there was no significant difference for psychological impact between infected staff in ALDs and doctors and nurses before and after their diagnosis (Tables 2, 3). In terms of the mental attitude toward the COVID-19 outbreak, no significant difference was also observed between uninfected and infected staff before diagnosis in ALDs. However, uninfected staff was fully aware of the seriousness of the epidemic compared with infected staff (Table 4).

**Table 2:** The psychological impact of infected staff members in administrative and logistics departments and doctors and nurses before diagnosis.

**Before diagnosis**	**Cases** **(*n* = 18)**	**Doctors and nurses** **(*n* = 18)**	***P***
Awareness	2.00 (2.00, 3.00)	1.50 (1.00, 2.00)	0.054
**MENTAL ATTITUDE**			
Neutral	12 (66.67%)	15 (83.33%)	0.443
Anxious	4 (22.22%)	4 (22.22%)	1.000
Pessimistic	0 (0.00%)	0 (0.00%)	1.000
Fearful	2 (11.11%)	0 (0.00%)	0.486
Full of fighting spirit	2 (11.11%)	0 (0.00%)	0.486
Optimistic	4 (22.22%)	2 (11.11%)	0.658

*Awareness: 1 is fully aware of the seriousness of the epidemic, and 5 is totally unaware of the seriousness of the epidemic*.

**Table 3:** The psychological impact of infected staff members in administrative and logistics departments and doctors and nurses after diagnosis.

**After diagnosis**	**Cases** **(*n* = 18)**	**Doctors and nurses** **(*n* = 18)**	***P***
Psychological stress	4.00 (3.00, 4.00)	4.00 (2.00, 5.00)	0.859
**THE POSSIBLE CAUSES**			
Disease related issues	9 (64.29%)	13 (81.25%)	0.417
Economic burden	2 (14.29%)	0 (0.00%)	0.209
Discrimination by others	4 (28.57%)	7 (43.75%)	0.466
Changes in the environment	3 (21.43%)	5 (31.25%)	0.689
Family member's health	14 (100.00%)	13 (81.25%)	0.228
Negative news via the internet	8 (57.14%)	8 (50.00%)	0.730
Others	0 (0.00%)	1 (6.25%)	1.000
**THE METHODS OF CONTROLLING STRESS**			
Communicate with others on WeChat	9 (50.00%)	10 (55.56%)	1.000
Video call with family or colleagues	10 (55.56%)	11 (61.11%)	1.000
Seek professional psychological crisis intervention	3 (16.67%)	3 (16.67%)	1.000
Read recent literature on this disease	2 (11.11%)	6 (33.33%)	0.229
Self-control	14 (77.78%)	14 (77.78%)	1.000
Avoid considering information about this outbreak	3 (16.67%)	3 (16.67%)	1.000
Others	1 (5.56%)	0 (0.00%)	1.000
**SOURCES OF COMFORT AND CARE AVAILABLE**			
Partner (lover)	13 (72.22%)	16 (88.89%)	0.402
Parents	9 (50.00%)	9 (50.00%)	1.000
Children	10 (55.56%)	5 (27.78%)	0.176
Brothers and sisters	9 (50.00%)	4 (22.22%)	0.164
Leaders and colleagues	15 (83.33%)	15 (83.33%)	1.000
News reports	2 (11.11%)	2 (11.11%)	1.000
Counseling professional or general	3 (16.67%)	0 (0.00%)	0.229

**Table 4:** The psychological status of infected and uninfected staff members in administrative and logistics departments.

**Psychology**	**Infected** **(*n* = 18)**	**Uninfected** **(*n* = 18)**	***P***
Awareness	2.00 (2.00, 3.00)	1.00 (1.00, 1.00)	0.007
**MENTAL ATTITUDE**			
Neutral	12 (66.67%)	11 (61.11%)	1.000
Anxious	4 (22.22%)	6 (33.33%)	0.711
Pessimistic	0 (0.00%)	2 (11.11%)	0.486
Fearful	2 (11.11%)	3 (16.67%)	1.000
Full of fighting spirit	2 (11.11%)	1 (5.56%)	1.000
Optimistic	4 (22.22%)	3 (16.67%)	1.000

*Awareness: 1 is fully aware of the seriousness of the epidemic, and 5 is totally unaware of the seriousness of the epidemic*.

## Discussion

It has been reported that the infection rates among healthcare workers during SARS and Middle East Respiratory Syndrome (MERS) were 20 and 26%, respectively (Al-Tawfiq and Memish, 2019). Unfortunately, the SARS-CoV-2 also infected a large number of healthcare workers. During previous infectious diseases outbreaks, studies on the healthcare staff's infection have focused on the front-line doctors and nurses, while the staffs in ALDs were often ignored. These staff are crucial to the normal operation of the hospital, so protecting this population from infection is also crucial to success in fighting COVID-19.

Our study included all 18 infected staffs in ALDs in Zhongnan Hospital of Wuhan University: 27.78% were in the logistics support department and therefore regularly come into contact with medical wastes when cleaning the hospital. There is no air circulation in the overcrowded elevator, and the infection of the elevator operator will thus expose all occupants to the virus. A recent study indicated that both air and surfaces may be contaminated by SARS-CoV-2; we therefore suggest that these staff must wear gloves and masks correctly in their routine work (Ong et al., 2020). For departments that have contact with doctors and nurses, such as Personnel, Finance and Operation Management Departments, one infected staff member may transmit the virus to other staff and cause explosive infection both in the same department and also in clinical departments, and this potentially causes nosocomial infection. Hence, special windows should be set up, and gloves and masks should be worn when documents are submitted and collected, especially in the autumn and winter when infectious diseases are prone to occur. Additionally, hospitals can adopt the paperless offices in ALDs, thereby reducing direct and indirect contact with potentially contaminated materials. Staff in the Sterile Supply Department should wear more advanced protective equipment when cleaning surgical instruments and transporting patients (Suen et al., 2020). Staff in Convalescent Department should take the same protective measures as healthcare workers when visiting patient's home, as they may constitute an infection source to spread the virus to other ALDs and clinical departments. At the same time, we should pay attention to the disinfection of offices, and careful use of central air conditioning in ALDs.

The emerging virus outbreaks have had a significant psychological impact on healthcare workers. Several viral outbreaks have occurred in the past 20 years, such as SARS, MERS, and Ebola disease (Kisely et al., 2020), and previous studies have reported that doctors and nurses at the frontline involving diagnosis and treatment commonly reported psychological problems during SARS epidemic in 2003 (Bai et al., 2004; Lee et al., 2007) and the MERS outbreak of 2014 (Lee et al., 2018). Currently, several published studies highlighting psychological effects on healthcare workers during the COVID-19 outbreak indicate that healthcare workers are at increased risk of psychological distress (Jin et al., 2020a; Kisely et al., 2020). Most studies about the psychological impact focused on doctors and nurses who performed the tasks of diagnosis and treatment, while few studies have been conducted on the psychological effects of the COVID-19 epidemic on staff in ALDs who were not infected (Chen et al., 2020; Huang et al., 2020a,b; Lai et al., 2020; Xing et al., 2020). However, no studies have focused on the psychological state of staff in hospitals who have been infected. Thus, it can be argued that our study is of great significance for further understanding the psychological effects on staff in ALDs during virus epidemics. Our study indicated most of staff in ALDs experienced psychological stress or emotional changes. A total of 50% of them were anxious about their conditions due to a lack of professional knowledge. Almost everyone was concerned about health of his/her family members' health, and eight staff members were influenced by negative news via the internet. Additionally, most logistics staffs are not regular employees of the hospital, and they may thus suffer from the risk of unemployment due to the impact of the epidemic, which further increases their psychological burden. Consequently, psychological intervention treatment is very urgent to cope with the psychological stresses and emotional changes among this group of staff.

In our study, we found no significant difference in psychological impact between infected doctors and nurses and staff in ALDs—neither in the awareness and mental attitude to the epidemic before diagnosis or the psychological changes after diagnosis. The results indicated that working in hospital and having clinical professional knowledge does not affect the psychological impact of COVID-19 epidemic on hospital staff. In ALDs, the mental attitude to the epidemic was not different between infected and uninfected staff, while uninfected staff members' awareness of the epidemic was higher than that of infected staff, which may have reduced the risk of infection by influencing their behavior. The main limitation of this study is that it was a single-center study with a small sample size. Although all infected staff in ALDs in this hospital were included, more studies are needed to verify the results. Additionally, some memory bias maybe exist among participates.

In conclusion, reasonable effective protective measures should be taken for staff in ALDs, such as setting up specialized windows for departments that have prolonged contact time with healthcare workers, adopting paperless offices to reduce contact with potentially contaminated materials, choosing appropriate protective equipment, disinfecting offices properly, and using central air conditioning carefully. Most staff experienced psychological stress during their isolation period after diagnosis, and psychological interventions are thus very urgent when it comes to coping with psychological distress among this group of people. Verification is needed using multi-center studies with a larger sample size in the future.

## Data Availability Statement

All datasets presented in this study are included in the article/supplementary material.

## Ethics Statement

The studies involving human participants were reviewed and approved by Committee for Ethical Affairs of Zhongnan Hospital of Wuhan University. The patients/participants provided their written informed consent to participate in this study.

## Author Contributions

X-TZ, X-HW, and LC: The conception and design of the study. LC, L-SL, Y-HJ, Z-YP, and X-TZ: Collection and assembly of data. L-SL, Y-HJ, and X-TZ: Analysis and interpretation of the data and Drafting the article. Y-HJ, LC, Z-YP, X-TZ, and X-HW: Revising it critically for important intellectual content. All authors contributed to the article and approved the submitted version.

## Conflict of Interest

The authors declare that the research was conducted in the absence of any commercial or financial relationships that could be construed as a potential conflict of interest.
